# Identification of the *Acinetobacter baumannii* Ribonuclease P Catalytic Subunit: Cleavage of a Target mRNA in the Presence of an External Guide Sequence

**DOI:** 10.3389/fmicb.2018.02408

**Published:** 2018-10-08

**Authors:** Carol Davies-Sala, Saumya Jani, Angeles Zorreguieta, Marcelo E. Tolmasky

**Affiliations:** ^1^Center for Applied Biotechnology Studies, College of Natural Sciences and Mathematics, California State University, Fullerton, Fullerton, CA, United States; ^2^Fundación Instituto Leloir, IIBBA-CONICET, Buenos Aires, Argentina; ^3^Facultad de Ciencias Exactas y Naturales de la Universidad de Buenos Aires, University of Buenos Aires, Buenos Aires, Argentina

**Keywords:** RNase P, Acinetobacter, ESKAPE, ribozyme, EGS technology, antisense

## Abstract

The bacterial ribonuclease P or RNase P holoenzyme is usually composed of a catalytic RNA subunit, M1, and a cofactor protein, C5. This enzyme was first identified for its role in maturation of tRNAs by endonucleolytic cleavage of the pre-tRNA. The RNase P endonucleolytic activity is characterized by having structural but not sequence substrate requirements. This property led to development of EGS technology, which consists of utilizing a short antisense oligonucleotide that when forming a duplex with a target RNA induces its cleavage by RNase P. This technology is being explored for designing therapies that interfere with expression of genes, in the case of bacterial infections EGS technology could be applied to target essential, virulence, or antibiotic resistant genes. *Acinetobacter baumannii* is a problematic pathogen that is commonly resistant to multiple antibiotics, and EGS technology could be utilized to design alternative therapies. To better understand the *A. baumannii* RNase P we first identified and characterized the catalytic subunit. We identified a gene coding for an RNA species, M1_Ab_, with the expected features of the RNase P M1 subunit. A recombinant clone coding for M1_Ab_ complemented the M1 thermosensitive mutant *Escherichia coli* BL21(DE3) T7A49, which upon transformation was able to grow at the non-permissive temperature. M1_Ab_ showed *in vitro* catalytic activity in combination with the C5 protein cofactor from *E. coli* as well as with that from *A. baumannii*, which was identified, cloned and partially purified. M1_Ab_ was also able to cleave a target mRNA in the presence of an EGS with efficiency comparable to that of the *E. coli* M1, suggesting that EGS technology could be a viable option for designing therapeutic alternatives to treat multiresistant *A. baumannii* infections.

## Introduction

**FIGURE 1:**
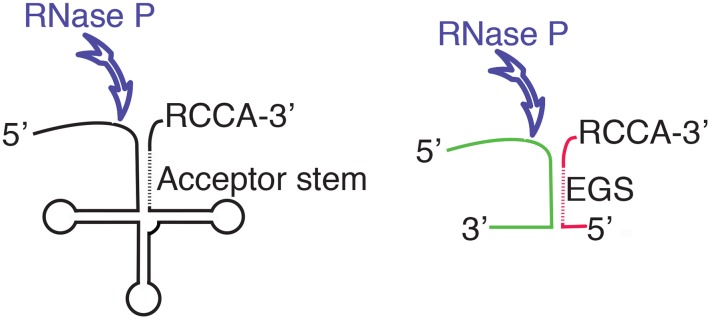
Diagram showing the structure of the pre-tRNA and the location of the endonucleolytic cleavage by RNase P (left). The diagram to the right shows a complex between two RNA molecules with a complementary region that form the appropriate structure to be recognized as substrate by RNase P. The different colors illustrate the fact that while there are structural requirements, RNase P does not show specificity of sequence. The antisense regions in the acceptor stem and the EGS are shown as dashed lines. The RCCA sequence facilitates the interaction between the substrate and RNase P.

Ribonuclease P, or RNase P, is a ubiquitous ribozyme that was first identified for its participation in the maturation of the precursor tRNA (pre-tRNA) by endonucleolytic cleavage at the 5′-end of the molecule (Robertson et al., 1972). Later, it was shown that RNase P participates in other biological processes like the synthesis of other RNA species such as transfer messenger RNA, precursors to 4.5S RNA, some multicistronic mRNAs, small non-coding RNA genes, phage-related RNAs, and others (Bothwell et al., 1976; Alifano et al., 1994; Komine et al., 1994; Hartmann et al., 1995; Reiner et al., 2006; Yang and Altman, 2007; Jarrous and Gopalan, 2010; Altman, 2011; Klemm et al., 2016). The RNase P holoenzyme is a ribonucleoprotein composed by the RNA molecule responsible for its catalytic activity, known as M1 in *Escherichia coli* (M1_Ec_), and one or more proteins that act as cofactors (Guerrier-Takada et al., 1983; Marvin and Engelke, 2009; Jarrous, 2017). In particular, bacterial RNase P holoenzymes usually contain only one small cofactor protein, called C5 in *E. coli* (C5_Ec_) (Reiter et al., 2010; Mondragon, 2013). Numerous studies on the RNase P enzymes from different organisms belonging to all three life domains showed many common structural features among the RNA components and a common core with similar secondary structure (Chen and Pace, 1997; Mondragon, 2013). Three types of RNA components of RNase P were identified in bacteria, the most common are known as types A (for ancestral) and B (for *Bacillus*); type C includes RNA molecules from green non-sulfur bacteria (Haas and Brown, 1998). For a comparative structural diagram among all three types see the review by Mondragon (Mondragon, 2013). The RNA components of bacterial RNase P include two domains, one of them called C (catalytic) that recognizes the acceptor stem and the 3′-CCA sequence of the substrate RNA and mediates the endonucleolytic cleavage. The other domain is called S (specificity) and is responsible for substrate recognition (Mondragon, 2013). Crystallographic studies on bacterial RNase P are limited, only the three-dimensional structures of the intact *Thermotoga maritima* RNA component and a fragment including the C domain of that of *Bacillus stearothermophilus* are available (Kazantsev et al., 2005; Torres-Larios et al., 2005). However, although not at the structural level, the *E. coli* RNase P, which consists of the 377-nucleotides catalytic RNA subunit M1_Ec_ and the 119-amino acids cofactor protein C5_Ab_, is one of the best characterized (Guerrier-Takada et al., 1983). RNase P requires a particular structure in the substrate RNA that includes a double-stranded region (acceptor stem) followed by a single-stranded stretch that includes the RCCA sequence at the 3′ end, which facilitates interaction with the enzyme (**Figure 1**). One of the two complementary segments of the acceptor stem is called “external guide sequence” (EGS) and is instrumental in guiding RNase P to cleave the opposite strand during the maturation process (**Figure 1**). With the exception of the acceptor stem, most other regions of the substrate RNA can be deleted without completely abolishing RNase P activity and, although essential for cleavage, the EGS is not required to be tethered to the rest of the molecule (Forster and Altman, 1990; Gopalan et al., 2002; **Figure 1**). Furthermore, cleavage is not dependent on the sequence of the substrate (**Figure 1**). This finding was the foundation of the EGS technology, a gene silencing strategy in which a short oligomer (EGS) interacts with a target RNA, usually mRNA, and elicits its cleavage, interfering with expression of the gene (Lundblad and Altman, 2010). EGS Technology approaches have been explored as alternatives to design therapeutic tools or antibiotic adjuvants for treatment of multidrug resistant bacterial infections (Guerrier-Takada et al., 1997; Soler Bistue et al., 2007, 2009; Ko et al., 2008; Shen et al., 2009; Sawyer et al., 2013).

*Acinetobacter baumannii* is a nosocomial pathogen that belongs to the ESKAPE group of pathogens and causes a wide range of severe infections, mainly among immunocompromised patients (Wong et al., 2017; Harding et al., 2018). Treatment of these infections is complicated by the multidrug-resistant nature of most strains, a characteristic that led to inclusion of *A. baumannii* in the priority list of antibiotic-resistant bacteria by the World Health Organization (Doi et al., 2017; Tacconelli et al., 2018; Tacconelli and Magrini, 2018). As a consequence, there is a high need for new treatments of *A. baumannii* and development of EGSs that inhibit expression of essential functions or antibiotic-resistance genes could be an alternative (Sala et al., 2012). A better understanding of the *A. baumannii* RNase P will be beneficial for EGS Technology development of therapies to treat multidrug resistant infections caused by this bacterium.

## Materials and Methods

### Bacterial Strains and Plasmids

*Acinetobacter baumannii* ATCC 17978 (Smith et al., 2007) was used as source of genomic DNA and the primers were designed using the complete genome sequence (Accession No. CP000521.1). *E. coli* DH5α (Taylor et al., 1993) and BL21(DE3) (Studier et al., 1990) were used as hosts for cloning experiments. *E. coli* BL21(DE3) T7A49 is a thermosensitive M1 mutant (Guerrier-Takada et al., 1995). Plasmid pM1Ab was generated by inserting a DNA fragment including the *A. baumannii rnpB* gene (*rnpB*_Ab_) under the control of the T7 promoter into the cloning vector pCR2.1 (Life Technologies). This fragment was an amplicon generated using *A. baumannii* ATCC 17978 chromosomal DNA as template and the primers 5′-GCAAGCTTTAATACGACTCACTATAGGGGAAGTGAGCCGGATGGTC-3′ (the T7 promoter is underlined) and 5′-GCAGGATCCAGGTGAAGTGAGCCTATAAGCC-3′. The sequence of EGSA2 was 5′-CGAUAUGAGAUCGACCA-3′. Plasmid pC5Ab was generated by ligating an amplicon including the *A. baumannii rnpA* gene (*rnpA*_ab_) to *Nde*I and *Xho*I-digested pET22(+) (Novagen). The amplicon was obtained using the primers 5′-GCCCATATGGTGCATCAACCCCATTTTTT-3′ and 5′-GAACTCGAGATTCTGCGAGGTTGGGACA-3′. The recombinant plasmid pC5Ab codes for the *A. baumannii* C5 (C5_Ab_) protein fused to a His-tag at the C-terminus under the control of the T7 promoter.

### General Procedures

Plasmid DNA preparations were carried out using the Wizard^®^ Plus SV Minipreps DNA Purification System (Promega). Endonuclease restriction and ligase treatments were performed according to the recommendations of the supplier (New England Biolabs). Polymerase chain reactions were carried out using Taq DNA Polymerase (Invitrogen) and the primers indicated (purchased from IDT Technologies), following the supplier’s recommendations. *In vitro* synthesis of RNA molecules was done using a MEGAshortscript high-yield transcription T7 kit according to the protocols provided by the supplier (Ambion). Denaturing polyacrylamide gel electrophoresis was performed as described previously (Sarno et al., 2003) on 6% polyacrylamide 19:1 (acrylamide–bis-acrylamide), gels containing 7 M urea using a Tris-Borate-EDTA buffer (TBE) or glycerol-tolerant gel (GTG) buffer. Electrophoresis of an aliquot of the M1_Ab_ purified transcript is shown in **Supplementary Figure S1A**. DNA and RNA sequence analyses were carried out using Basic local alignment search tool (BLAST) (Altschul et al., 1990), MUSCLE (Edgar, 2004), and Bcheck (Yusuf et al., 2010). *E. coli* M1 (M1_Ec_) and C5 (C5_Ec_) were purified as described before (Guerrier-Takada et al., 1983).

Overexpression of C5_Ab_ was carried out culturing *E. coli* BL21 (DE3; pC5Ab) in LB broth containing 100 μg of ampicillin/ml at 37°C until the optical density at 600 nm (OD600) was 0.8. At this point isopropyl β-D-galactopyranoside (IPTG) was added to a concentration of 0.5 mM, the culture was incubated for 90 m at 28°C, and the cells were harvested by centrifugation and suspended in phosphate buffer saline (PBS) buffer (20 mM sodium phosphate and 300 mM sodium chloride) pH 7.4. Cells were lysed by French press at 1000 psi (three passages) followed by centrifugation at 4000 *g* at 4°C for 15 min to remove cell debris. The supernatant was centrifuged at 13500 *g* at 4°C for 20 min and the supernatant was subjected to one last centrifugation at 100000 *g* for 1 h at 4°C. The supernatant containing the soluble fraction was used for purification of C5_Ab_. The protein was purified by immobilized metal affinity chromatography (IMAC) using a nickel-charged nitrilotriacetic acid (NTA) resin column (Thermo Scientific HisPur Ni-NTA Spin Columns) following the recommendations of the supplier. Once eluted using PBS containing 250 mM imidazole and 6 M Guanidine HCl, pH 7.4, the samples were dialyzed against PBS at 4°C. Proteins were analyzed using sodium dodecyl sulfate 15% polyacrylamide gel electrophoresis (SDS-PAGE)(Laemmli, 1970) followed by staining with Coomassie Brilliant Blue. Nucleotide and amino acid sequence analyses and comparisons were performed using the Clustal Omega and the BLAST programs (Altschul et al., 1990; Sievers et al., 2011). The prediction of physical and chemical parameters, search of specific domains, and modeling were carried out using the ProtParam, Pfam, and SWISS-MODEL, respectively (Gasteiger et al., 2005; Finn et al., 2014; Bienert et al., 2017). An SDS-PAGE analysis of the purified C5_Ab_ protein is shown in **Supplementary Figure S1B**.

### *In vitro* RNase P Assays

Unimolecular substrate: the reaction contained pre-tRNA^Tyr^ (80 pmol), M1_Ec_ or M1_Ab_ (40 pmol), C5_EC_ or C5_Ab_ (50 pmol; when indicated) in C5 buffer (20 mM HEPES-KOH pH = 8, 400 mM ammonium acetate, 10 mM magnesium acetate, 5% glycerol) in a total volume of 10 μl. Incubation was performed at 37°C for 90 m. Bimolecular substrate: *aac(6)′-Ib* mRNA (40 pmol) was incubated with EGS (50 pmol) at room temperature for 15 min. Simultaneously, M1_Ab_ (40 pmol) was incubated with C5_Ec_ (50 pmol) in C5 buffer at 37°C for 15 min. Both fractions were combined and incubated for 90 min at 37°C. The reaction was stopped by heating and subjected to phenol/chloroform extraction followed by ethanol precipitation as described before (Jani et al., 2018). The products were resuspended in 1 volume of gel loading buffer and analyzed by 6% denaturing TBE-PAGE or GTG-PAGE (Soler Bistue et al., 2009). RNA bands were visualized by staining with ethidium bromide and UV transillumination.

### M1 Heterologous Complementation Assays

*Escherichia coli* BL21(DE3) T7A49 and *E. coli* BL21(DE3) T7A49 (pM1Ab) were incubated in LB broth for 24 h at 28 or 42°C, with or without 0.1 mM IPTG. Bacterial growth was determined by measuring OD_600_. Assays were carried out in duplicate and repeated three times. Statistical significance was analyzed by one-way ANOVA with Dunnett’s multiple comparison test. *P* < 0.05 was considered statistically significant.

## Results and Discussion

**FIGURE 2:**
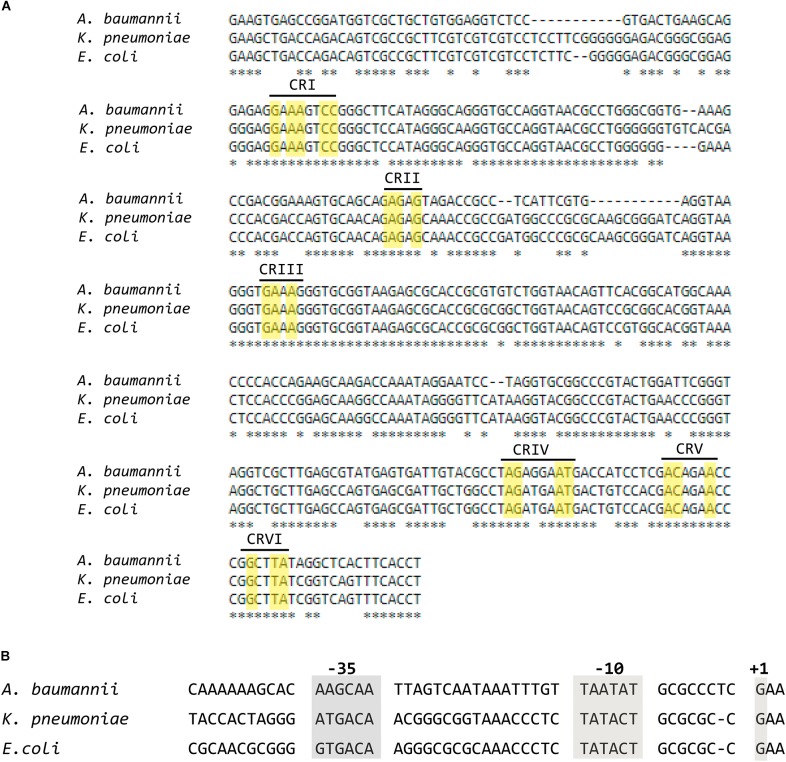
Alignment of the nucleotide sequences of the coding and promoter regions of RNA subunit (M1) of the ribonuclease P from *Escherichia coli*, *Klebsiella pneumoniae*, and *Acinetobacter baumannii*. **(A)** Multiple alignment of the coding sequences of M1 performed using MUSCLE. The yellow rectangles show nucleotides belonging to the universally Conserved Regions present in RNA subunits of RNase P (Chen and Pace, 1997), each region is indicated in roman numbers. **(B)** Alignment of the promoter regions.

A BLAST search using as query the *E. coli rnpB* nucleotide sequence (Accession No. NCBI Gene ID 947634) and the complete genome of *A. baumannii* ATCC 17978 as subject (Accession No. GenBank: CP000521.1) identified a region (coordinates 987928–988235) with 80% identity to the *E. coli rnpB* that was called *rnpB*_Ab_. A comparative analysis of the nucleotide sequence of this region to the *rnpB* genes from *E. coli* and *Klebsiella pneumoniae* (Accession No. GenBank: M32719.1) (Lawrence et al., 1987) permitted us to determine the *rnpB*_Ab_ promoter region as well as the first and last nucleotide of the RNA molecule encoded, called M1_Ab_ (**Figure 2A**). Inspection of the M1_Ab_ sequence shows that it belongs to the type A group of RNase P catalytic subunits and includes the conserved regions (Chen and Pace, 1997; Mondragon, 2013). Further analysis using Bcheck (Yusuf et al., 2010) identified the M1_Ab_ sequence as an RNase P RNA. The high level of identity between the sequence of *rnpB*_Ab_ and those from the *rnpB* genes from *E. coli* and *K. pneumoniae* extends from the -10 nucleotide in the promoter region (Lawrence et al., 1987) to the 3′ end of the RNA molecule. Conversely, the -35 and spacer regions show divergence (**Figure 2B**). This could be a consequence of adaptation to different properties between the RNA polymerases from *A. baumannii* and the two Enterobacteriaceae. Future comparative studies of expression and activity levels in all three bacteria may lead to a better understanding of the significance of these differences.

**FIGURE 3:**
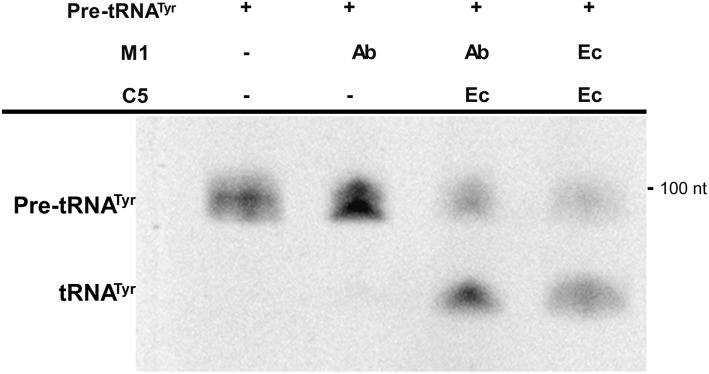
*In vitro* activity of the RNA subunit of the ribonuclease P from *Acinetobacter baumannii* (M1_Ab_). The unimolecular substrate pre-RNAt^Tyr^, was used for the reaction performed as described in section “Materials and Methods”. The reaction was analyzed on denaturing PAGE. The location and size of the substrate and the cleavage product are indicated to the left and right, respectively. Ab, *A. baumannii* and Ec, *E. coli*. C5

**FIGURE 4:**
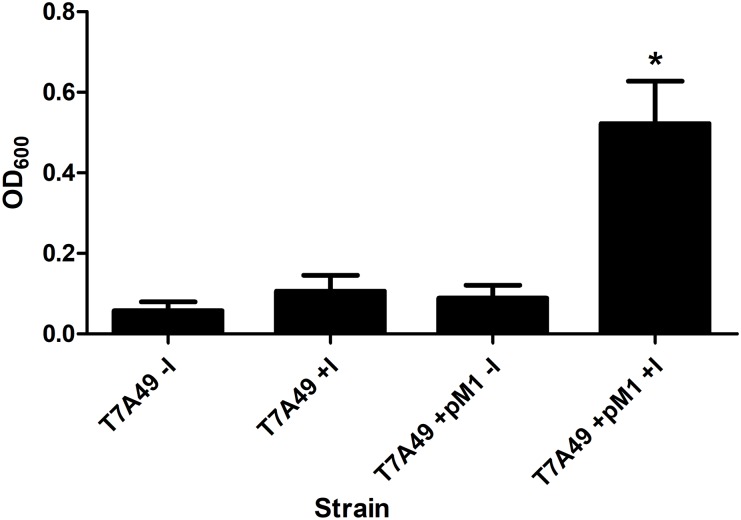
Heterologous complementation of strain *E. coli* BL21(DE3) T7A49. The M1_EC_ thermosensitive mutant was transformed with pM1Ab, a plasmid encoding M1_Ab_ under the control of a T7 promoter. *E. coli* BL21(DE3) T7A49 (T7A49) and *E. coli* BL21(DE3) T7A49 (pM1Ab) (T7A49M1) were cultured in the presence (IPTG) or absence of 0.1 mM IPTG Growth was assessed measuring OD_600_. Statistical analysis was carried out using one-way ANOVA and Dunnett’s multiple comparison test. ^∗^indicates statistical significance (*P* < 0.05).

The *rnpB*_Ab_ gene was cloned under the control of the T7 promoter and the 355-nt M1_Ab_ RNA was synthesized *in vitro* as described in section “Materials and Methods” (**Supplementary Figure S1A**). The synthesized product was tested to determine its RNase P activity using as substrate pre-tRNA^Tyr^. **Figure 3** shows that both M1_Ab_ and the M1_Ec_ cleaved the substrate with similar efficiency in the presence of the cofactor protein C5_Ec_ and were inactive in the absence of the protein in the conditions used in the assay. Previous work carried out with M1 showed that at certain magnesium concentrations *in vitro*, cleavage occurs in the absence of C5 (Guerrier-Takada et al., 1983). The results of the experiment shown in **Figure 3** not only confirmed that the M1_Ab_ RNA is the *A. baumannii* RNase P catalytic subunit, but also that it is active in the presence of a heterologous cofactor as it is the C5_Ec_ protein. To confirm the activity of M1_Ab_ *in vivo*, we carried out an experiment using the M1 thermosensitive mutant *E. coli* BL21(DE3) T7A49, which does not grow at the non-permissive temperature (42°C). This strain was transformed with the plasmid pM1Ab and the transformant strain was cultured at 28 and 42°C in the presence or absence of IPTG. **Figure 4** shows that the *E. coli* BL21(DE3) T7A49(pM1Ab) acquired the ability to grow at 42°C when expression of M1_Ab_ was induced by addition of IPTG, indicating that RNase P function was restored. This result showed that, as it was the case for the *in vitro* reaction, M1_Ab_ could interact with C5_Ec_ and produce a functional RNase P *in vivo*.

**FIGURE 5:**
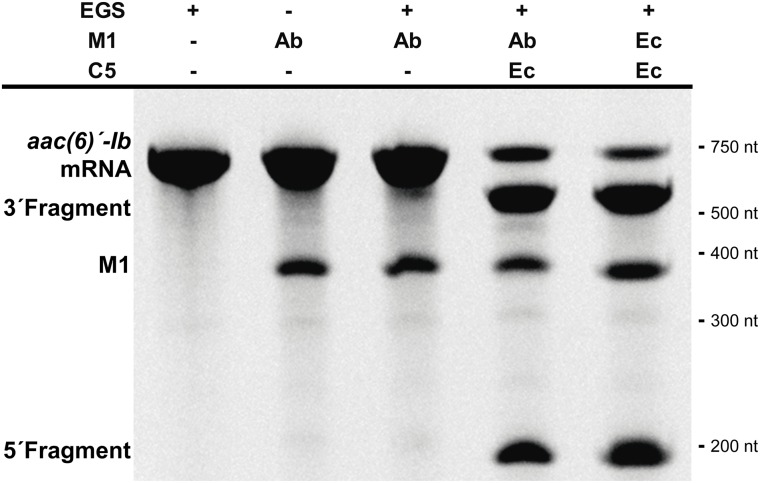
*Acinetobacter baumannii* RNase P RNA subunit and its application for EGS technology. *In vitro* activity of the M1_Ab_, RNA subunit of ribonuclease P of *A. baumannii* in presence of a bimolecular substrate. The substrate is composed of mRNA *aac(6)′-Ib* and EGSA2. The reaction was analyzed on denaturing PAGE. On the left is shown the location of the substrate, M1Ab and cleavage products. Molecular size standards are shown to the right. Ab, *A. baumannii* and Ec, *E. coli*. C5

Studies on the RNase P showed that most of the pre-tRNA substrate molecule could be removed without affecting its activity (Gopalan et al., 2002; Lundblad and Altman, 2010). Furthermore, bimolecular complexes were also substrates as long as they form the appropriate structure regardless of the nucleotide sequence (Gopalan et al., 2002; Lundblad and Altman, 2010). These findings originated what is known as EGS technology, which takes advantage of the host RNase P activity to induce degradation of a target mRNA in the presence of an antisense oligonucleotide known as EGS (Gopalan et al., 2002; Lundblad and Altman, 2010; Davies-Sala et al., 2015). This technology could be an option for designing antimicrobials that target essential *A. baumannii* functions or adjuvants that inhibit expression of resistance genes and would be used in combination with the appropriate antibiotic to restore its therapeutic power. We assessed the ability of M1_Ab_ to elicit cleavage of a target mRNA in the presence of an EGS (bimolecular RNA substrate) in comparison to that of M1_EC_. For this we used a bimolecular substrate consisting of the *aac(6’)-Ib* mRNA, which codes for an acetyltransferase that catalyzes inactivation of several aminoglycosides of clinical relevance (Ramirez and Tolmasky, 2010; Ramirez et al., 2013), and an EGS, EGSA2, that elicits cleavage of the mRNA by the *E. coli* RNase P holoenzyme (Soler Bistue et al., 2009). **Figure 5** shows that the reactions carried out with both M1_EC_ and M1_Ab_ produced the same level of degradation of the *aac(6’)-Ib* mRNA strongly suggesting that EGS technology could be an alternative for novel treatments of *A. baumannii* infections.

**FIGURE 6:**
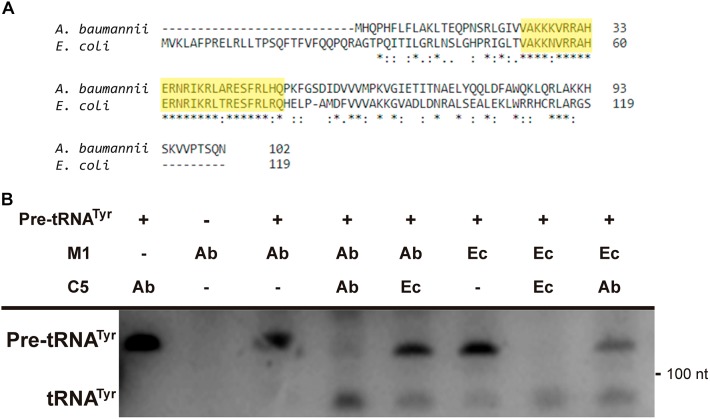
C5_Ab_ protein. **(A)** C5_Ec_ and C5_Ab_ amino acid sequence comparison. **(B)** *In vitro* activity of the reconstituted RNase P holoenzymes. The substrate pre-RNAt^Tyr^ was incubated with the subunits indicated on top in the conditions described in section “Materials and Methods”. The reaction was analyzed on denaturing PAGE, the fourth and fifth wells were run in the same gel but were not contiguous to the first three. The location and size of the substrate and the cleavage product are indicated to the left and right, respectively. Ab, *A. baumannii* and Ec, *E. coli*.

Further analysis of the *A. baumannii* ATCC17978 genome sequence permitted us to identify an open reading frame potentially coding for C5_Ab_, the *A. baumannii* RNase P cofactor protein. Amino acid sequence comparison between the C5_Ec_ and C5_Ab_ proteins showed low similarity throughout most of the sequence. However, a shared conserved 30-amino acid central core characteristic of C5 proteins was identified (**Figure 6A**, highlighted in yellow). The C5_Ab_ predicted isoelectric point was 10.8, characteristic of nucleic acids-binding proteins. Pfam analysis predicted this protein to possess a domain (amino acids 2–86) corresponding to cofactors of RNase P family proteins. The C5_Ab_ protein was used to reconstitute the *A. baumannii* holoenzyme. **Figure 6B** shows that M1_Ab_ or M1_Ec_ were activated in the presence of C5_Ab_ when tested using pre-tRNA^tyr^ as substrate.

In conclusion, the results described in this study indicate that we identified the *A. baumannii* ATCC 17978 RNase P gene coding for the catalytic subunit, M1_Ab_, and showed that its activity is comparable to that of the *E. coli* M1 subunit. M1_Ab_ was functional in the presence of C5_Ec_ as well as C5_Ab_, the latter of which was partially purified after the gene was identified and cloned. Furthermore, the M1_Ab_ ability to cleave otherwise non-substrate target mRNAs in the presence of an adequate EGS indicates that EGS technology could be a viable option for designing therapeutic alternatives to treat multiresistant *A. baumannii* infections. However, numerous challenges remain to be addressed before this technique can be reduced to practice. Non-hydrolyzable, but active analogs must be designed to ensure stability. Also, the compound must efficiently penetrate the cells once it reached the site of infection. Promising but still preliminary results have been obtained testing conjugates between nuclease resistant hybrid locked nucleic acids (LNA)/DNA oligomers and the cell penetrating peptide (RXR)_4_XB (where R stands for arginine, X for 6-aminohexanoic acid, and B for beta-alanine) (Jackson et al., 2016; Jani et al., 2018). Although previous reports indicate that antisense compounds containing LNA and DNA nucleotides show low toxicity (Wahlestedt et al., 2000), once a specific compound is identified as a candidate for treatment of *A. baumannii* infections, its cytotoxicity will have to be determined.

## Author Contributions

AZ, CD-S, and MT conceived and designed the experiments. CD-S and SJ performed the experiments. AZ, CD-S, MT, and SJ analyzed the data. AZ, CD-S, and MT wrote the paper.

## Conflict of Interest Statement

The authors declare that the research was conducted in the absence of any commercial or financial relationships that could be construed as a potential conflict of interest.
