# Supplementary material for: Identification of the Acinetobacter baumannii Ribonuclease P Catalytic Subunit: Cleavage of a Target mRNA in the Presence of an External Guide Sequence
Source: Front Microbiol. 2018 Oct 8;9:2408. doi: 10.3389/fmicb.2018.02408 (PMC6186949; doi:10.3389/fmicb.2018.02408)
Supplement: Supplementary file 1 [file Data_Sheet_1.docx]

**SUPPLEMENTARY MATERIAL**

**Identification of the *Acinetobacter baumannii* Ribonuclease P catalytic subunit: cleavage of a target mRNA in the presence of an external guide sequence**

Carol Davies-Sala^1,2,3♣^, Saumya Jani^1^, Angeles Zorreguieta^2,3^ and Marcelo E. Tolmasky^1#^

^1^Center for Applied Biotechnology Studies, College of Natural Sciences and Mathematics, California State University Fullerton, Fullerton, California, USA; ^2^Fundación Instituto Leloir, IIBBA-CONICET, Argentina; ^3^FCEyN UBA, University of Buenos Aires, Argentina


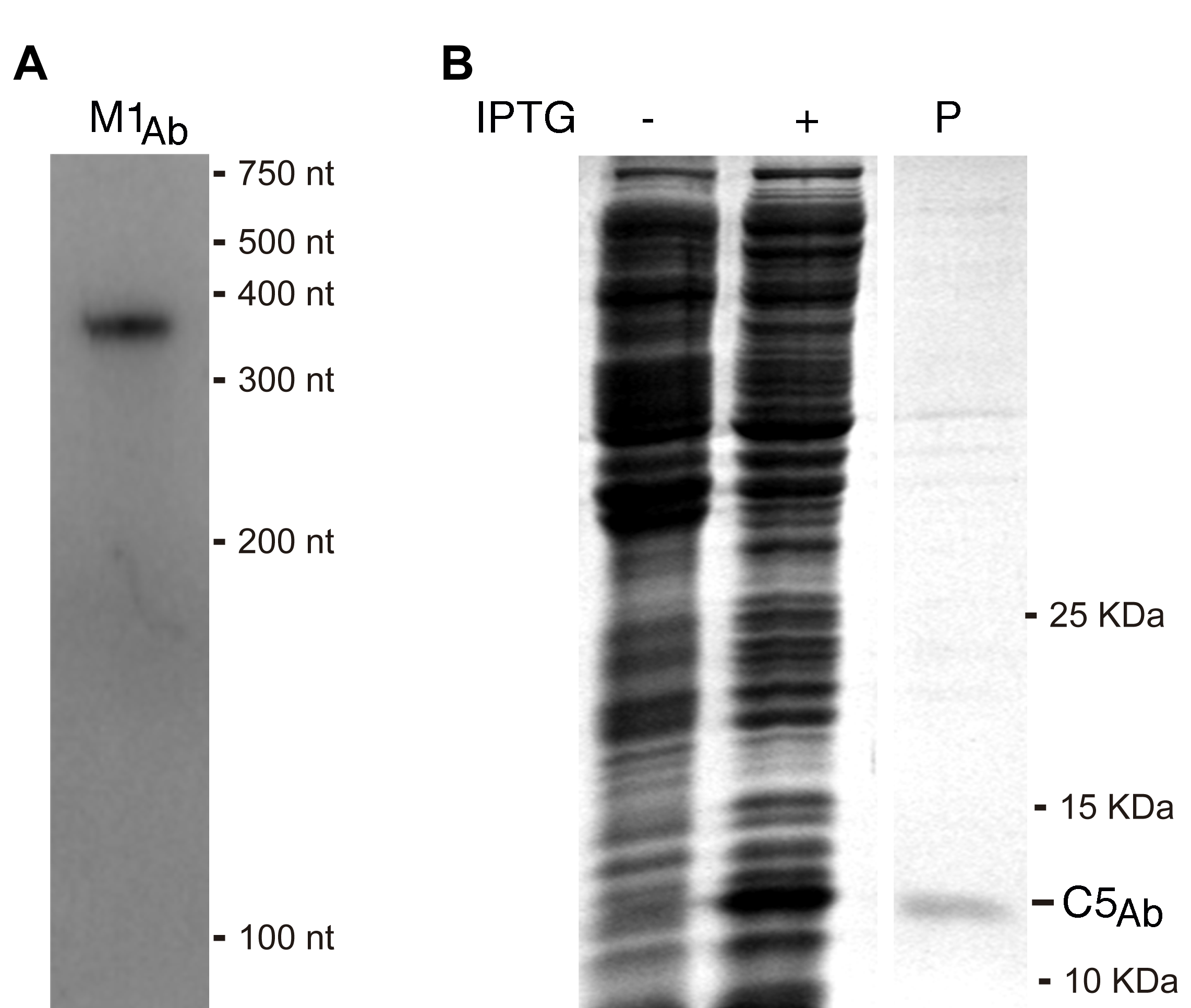


Fig. S1. A. In vitro transcription using as template plasmid pM1Ab. Transcription was carried out as described in Materials & Methods. The plasmid pM1Ab codes for the *A. baumannii* RNase P catalytic subunit (M1Ab). Molecular size standards are shown to the right. B. Expression and purification of the *A. baumannii* protein cofactor C5_Ab_. Extraction and purification were carried out as described in Materials & Methods. Soluble proteins from IPTG uninduced (-) and induced (+) *E. coli BL21*(DE3)(pC5Ab) cells; partially purified C5_Ab_ (P). Molecular weight standards are shown to the right.
